# Expression and prognostic significance of the m6A RNA methylation regulator HNRNPC in HNSCC

**DOI:** 10.3389/fonc.2025.1516867

**Published:** 2025-02-07

**Authors:** Yulin Zhang, Yixu Wang, Jilin Peng, Kun Zhao, Ling Li, Yuan Zhang, Ziyu Zhai, Sijie Yuan, Shichao Li, Fanglei Ye, Le Wang

**Affiliations:** ^1^ Department of Otolaryngology Head and Neck Surgery, The First Affiliated Hospital of Zhengzhou University, Zhengzhou, China; ^2^ Department of Otolaryngology, Head and Neck Surgery, People’s Hospital, Peking University, Beijing, China; ^3^ Department of Otolaryngology Head and Neck Surgery, Henan Provincial People’s Hospital, Zhengzhou, Henan, China; ^4^ Department of Otolaryngology Head and Neck Surgery, People’s Hospital of Zhengzhou University, Zhengzhou, Henan, China; ^5^ Department of Otolaryngology Head and Neck Surgery, People’s Hospital of Henan University, Zhengzhou, Henan, China

**Keywords:** heterogeneous nuclear ribonucleoprotein C, head and neck squamous cell carcinoma, prognostic significance, tumor progression, N6-methyladenosine

## Abstract

**Background:**

N6-methyladenosine (m6A) RNA modification is crucial for tumor development and progression; however, which m6A regulators play a pivotal role in head and neck squamous cell carcinoma (HNSCC) remains ambiguous.

**Methods:**

Utilizing the Cancer Genome Atlas (TCGA) database, the expression levels of m6A regulators in HNSCC were examined, which led to the identification of heterogeneous nuclear ribonucleoprotein C (*HNRNPC*) as a key gene. Further experiments were performed in patient samples, stable cell lines, and a murine xenograft tumor model.

**Results:**

A reliable survival risk model of m6A was constructed based on the TCGA database. Gene Expression Omnibus (GEO), normal and tumor tissue microarrays (TMA), and tumor tissue samples from patients with HNSCC were observed that a high level of *HNRNPC* expression was closely linked to a poor prognosis among patients. Knockdown of *HNRNPC* in the HNSCC cell lines HSC-3 and CAL-27 resulted in a significant decrease in proliferation, invasion, and malignant transformation abilities. RNA sequencing (RNA-seq) and methylated RNA immunoprecipitation and sequencing (MeRIP-seq) data revealed that *HNRNPC* is involved in cell differentiation, cell migration and apoptosis. The mouse xenograft model elucidated that *HNRNPC* can promote tumorigenesis and progression of HNSCC.

**Conclusions:**

*HNRNPC* can serve as a valuable predictor of tumor progression and prognosis in patients with HNSCC.

## Introduction

Head and neck squamous cell carcinoma (HNSCC) arises in the oropharynx, larynx, oral cavity, and hypopharynx and is the sixth most frequent type of malignant tumor worldwide (1). Patients are often diagnosed with advanced disease, which is more challenging to manage and may even be incurable. As a result, aggressive treatment options are often required, which can impact quality of life and may leave the patient functionally disabled (2). Despite significant advancements in treatment strategies over the past few decades (3), encompassing innovations in surgical procedures, radiation therapy, and cytotoxic drug therapies, recurrence and nodal metastasis persist in greater than 50% of HNSCC patients, emphasizing the low 5-year survival rate (1). Accurate prognostic assessment is therefore fundamentally important for the successful clinical management and shaping of personalized treatment choices for HNSCC.

N6-methyladenosine (m6A), methylation at the N6 position of adenosine, is the most prevalent modification in eukaryotes (4). The process of m6A modification is reversible (5) and consists of “writers” (METTL3, METTL14, and WTAP) with methyltransferase activity (6), “erasers” (FTO and ALKBH5) with demethyltransferase activity (7), and “readers” (YTHDC2, IGF2BP2, and HNRNPC) with methylation recognition function (8). The roles of m6A in the pathogenesis and progression of cancer have been confirmed in recent years (9). HNRNPC has been reported as the most common RNA binding protein (10) and one member of the m6A reader protein family (11), participating in RNA splicing (12), sequence-unspecific RNA exportation (13), RNA expression, RNA stability (14), RNA 3’ end processing (15), and translation (16). Increasing evidence highlights the aberrant upregulation of HNRNPC in a variety of tumors, which plays a non-negligible role in tumorigenesis and progression and affects overall survival in glioblastoma (17), hepatocellular carcinoma (18) and breast cancer (19). Despite extensive research and the analysis of innumerable HNRNPC profiles derived from cancer cells, the precise clinical prognostic significance of HNRNPC in HNSCC and its potential physiological functions within tumor cells are yet to be definitively understood. At present, there exists no comprehensive investigation on the role of HNRNPC in HNSCC.

We created a survival risk model using m6A-associated protein expression from the TCGA HNSCC dataset, and validated it with GEO database. Along with clinical HNSCC samples, showed that *HNRNPC* had the most noticeable difference in expression between tumor and normal samples. Knocking down *HNRNPC* in HNSCC cell lines HSC-3 and CAL-27 led to significant reductions in cell proliferation, invasion, and tumorigenesis. RNA-seq and MeRIP-seq data supported our findings, emphasizing the crucial role of HNRNPC in promoting malignant behavior in HNSCC cells. Targeting HNRNPC could potentially inhibit these cellular processes, offering a new avenue for improving patient prognosis and treatment outcomes.

## Materials and methods

### Patient samples

We selected 150 patients with head and neck squamous cell carcinoma (HNSCC) from the Department of Head and Neck Surgery and Stomatology at the First Affiliated Hospital of Zhengzhou University. Tissues removed during surgery were examined to confirm the diagnosis. The clinicopathological characteristics are shown in **Table 1**. Formalin-fixed and paraffin embedded (FFPE) tissues were used for qPCR and immunohistochemistry. Normal and tumor tissue microarrays (TMAs) were purchased from Servicebio Technology Co., Ltd., (Wuhan, China) which contained 45 cases of squamous carcinoma and 45 normal tissue samples. The present study was approved by the Ethics Committee of the First Affiliated Hospital of Zhengzhou University. Informed consent was waived due to the retrospective nature of tissue sample collection from patients diagnosed with HNSCC. Additionally, there exist no personal privacy or commercial interests.

**Table 1:** Comparison of the clinical characteristics between the low and high HNRNPC.

Variable	N of case	HNRNPC		*P*
		Low	High	
Sample	150	75	75	
Age(year)				**0.012**
≤60	91	38	53	
>60	59	37	22	
Gender				0.533
Male	139	71	68	
Female	11	4	7	
Tumor stage				0.971
T1	42	21	21	
T2	46	24	22	
T3	35	18	17	
T4	25	11	14	
Unknown	2	1	1	
Clinical stage				0.081
I	23	16	7	
II	25	15	10	
III	32	13	19	
IV	66	28	38	
Unknown	4	3	1	
Distant metastasis				0.316
Yes	1	0	1	
No	149	75	74	
Lymph node metastasis				**0.033**
Yes	81	34	47	
No	69	41	28	
Survival state				**0.02**
Alive	90	52	38	
Dead	60	23	37	

Bold values: P<0.05.

### Public mRNA expression datasets

RNA-seq data for HNSCC and normal samples were downloaded from the TCGA database. Level 3 TCGA RNA-seq data for 517 HNSCC samples (Illumina^®^ HiSeq 2000), with clinical annotations and overall survival (OS) information acquired from the Cancer Genomics Browser of University of California Santa Cruz (UCSC) (https://genomecancer.ucsc.edu), were used as the training set. A total of 253 primary cases, also with clinical annotations and OS information, were selected from the GSE65858 dataset (http://www.ncbi.nlm.nih.gov/geo).

### Cell culture

The HNSCC cell lines CAL-27 and HSC-3 were purchased from the Chinese Academy of Sciences typical culture preservation committee cell bank. Cells were maintained in Dulbecco’s modified Eagle’s medium (DMEM) (high glucose; Gibco) supplemented with 10% fetal bovine serum (FBS), 100 U/mL penicillin, and 100 mg/mL streptomycin (Invitrogen, Grand Island, NY, USA) and cultured in a humidified atmosphere of 5% CO_2_ at 37°C.

### ShRNA lentivirus production and infection

HEK293T cells were co-transfected with HNRNPC shRNA plasmids (Shanghai Genechem Co., Shanghai, China) and the lentivirus packaging plasmids psPAX2 and pmD2G using jetPRIME (Polyplus transfection Inc, Illkirch, France). At 6-h post transfection, the medium was replaced with DMEM (high glucose) containing 10% FBS. 48-h after transfection, viral particles were collected, and polybrene (8 µg/mL) was applied to assist in the infection of cells, from which GFP^+^ cells were obtained by flow cytometry. The RNAi Consortium (TRC) IDs for the shRNAs are: shHNRNPC-1: 5’-GCCTTCGTTCAGTATGTTAAT-3’ and shHNRNPC-2: 5’- GCGCTTGTCTAAGATCAAATT-3’. Scrambled shRNA was used as the negative control.

### RNA extraction and quantitative real-time PCR

Cellular RNA was extracted with TRIzol™ reagent (Invitrogen) and reverse- transcribed to complementary DNA (cDNA) using the High-Capacity cDNA Reverse Transcription Kit (Invitrogen). Quantitative PCR was carried out with the ABI StepOne™ Real-Time PCR system (Applied Biosystems) using SYBR^®^ Green Realtime PCR Master Mix (Toyobo). Relative gene expression was calculated using the 2 ^–ΔΔCt^ method. Primers were synthesized by Sangon Biotech (Shanghai, China): HNRNPC forward 5’-GTTACCAACAAGACAGATCCTCG-3’ and reverse 5’-AGGCAAAGCCCTTATGAACAG-3’; and GAPDH forward 5’-CAGGAGGCATTGCTGATGAT-3’ and reverse 5’-GAAGGCTGGGGCTCATTT-3’.

### Protein extraction and western blot

Cells were washed three times with PBS and lysed in RIPA (Beyotime, Guangzhou, China) buffer containing PMSF. Protein concentrations were quantitated using the BCA Protein Assay Kit (Beyotime, Guangzhou, China). Protein samples were separated by 8% or 10% SDS-PAGE and transferred to PVDF membrane (Millipore, Billerica, MA, USA). After blocking with 5% non-fat milk, the membranes were incubated overnight at 4°C with primary antibodies, followed by incubation with a secondary antibody at room temperature. The primary antibodies used were anti-hnRNPC (Abcam, ab133607, 1:1000) and anti-β-actin (Cell Signaling Technology, 3700, 1:1000). Protein–antibody complexes were detected with enhanced chemiluminescence (ECL) reagent (Bio-Rad, Hercules, CA) using the ChemiDoc™ XRS imaging system (Bio-Rad, USA).

### Immunohistochemistry

The patient tissues were fixed overnight with 10% (vol/vol) neutral-buffered formalin and embedded in paraffin. Sections of 5 µm thickness were then prepared. These sections were deparaffinized, rehydrated in xylene, and treated with graded ethanol concentrations. Endogenous peroxidase activity was quenched using 3% hydrogen peroxide, and antigen retrieval was carried out with an EDTA buffer. The sections were incubated overnight in a humid chamber at 4°C with the primary antibodies. After a 1-hour incubation with secondary antibodies at room temperature, the 3, 3’-diaminobenzidine (ZSGB-BIO, Beijing, China) detection system was used to visualize the staining. Stained tissue sections were observed using a light microscope (Nikon, Tokyo, Japan) and quantitatively scored based on the percentage of positive cells and staining intensity. The H-score was assessed using the following formula: (percentage of weak intensity ×1) + (percentage of moderate intensity ×2) + (percentage of strong intensity ×3).

### Cell proliferation assay

Cells were seeded on 96-well plates at a density of 1000 cells per well and incubated at 37°C, 5% CO_2_. The absorbance was measured on days 1, 2, and 3 at a wavelength of 450 nm (A_450_) using a microplate reader (Bio-Rad), after which CCK8 (Dojindo, Tokyo, Japan) was added and the cells were incubated for 3 h at 37°C. Cell proliferation was quantitated by measuring the relative cell viability.

### Cell apoptosis assay

Cells on 6-well plates were trypsinized, washed twice with cold PBS, and resuspended in 500 μL Annexin V binding buffer. After incubation with Annexin V-FITC and PI in the dark for 15 min, the cells were analyzed by flow cytometry, and the apoptotic population was measured using the FlowJo X software.

### Wound healing assay

Cells were seeded on 6-well plates at a density of 5 × 10^5^ cells/well, and at 90% confluence were scratched with a sterile pipette tip. After washing with culture medium to remove debris, cells were allowed to migrate for 48 h. The wounds were photographed at 0 h and 48 h using an Olympus IX53 inverted phase contrast microscope (Olympus, Tokyo, Japan). Three random fields were marked and measured. The migration index is expressed as the percentage of wound remaining at 48 h relative to that at 0 h.

### Migration and Matrigel^®^ invasion assay

Cells were resuspended in serum-free DMEM at a density of 5 × 10^4^ cells/mL. 200 μL diluted cells were seeded onto the upper compartment of Transwell^®^ chambers(8.0-μm pore size, Corning) or Matrigel^®^(5 × dilution, 50 μL, BD Bioscience), and 600 μL DMEM supplemented with 10% FBS was added to the lower compartment. After a 48-h incubation, the migrating/invading cells were fixed with 4% paraformaldehyde, stained with 0.1% crystal violet, photographed under a microscope, and then counted using the ImageJ software.

### RNA sequencing

SeqHealth Tech Co., Ltd. (Wuhan, China) conducted RNA extraction, library preparation, and high-throughput sequencing data analysis. The KCTM Stranded mRNA Library Prep Kit for Illumina^®^ (Wuhan Seqhealth Co., Ltd. China) was employed to prepare the RNA library, and 2 µg RNA per sample was used as input material. The library products corresponding to 200–500 bps were enriched, quantitated, and then sequenced and analyzed on the DNBSEQ-T7 sequencer (MGI Tech Co., Ltd. China) with the PE150 model. Gene Ontology (GO) enrichment analysis was performed using the KOBAS software (version: 2.1.1) to determine the biological implications of the differentially expressed genes (DEGs).

### Methylated RNA immunoprecipitation sequencing

MeRIP-seq data analysis was conducted by SeqHealth Tech Co., Ltd. (Wuhan, China). Total RNA was extracted by TRIzol™ reagent (Invitrogen), and contaminating DNA was digested with DNase I. A 50-μg aliquot of total RNA was subjected to polyadenylated RNA enrichment by VAHTS^®^ mRNA Capture Beads, and 20 mM ZnCl_2_ was used to shear the mRNA into approximately 100–200-nt fragments, which were then subjected to immunoprecipitation (IP) with an anti-m6A antibody (Synaptic Systems). The KC-Digital™ Stranded mRNA Library Prep Kit for Illumina^®^ (Wuhan SeqHealth Co., Ltd. China) was used to construct the stranded RNA sequencing library. The library products were enriched, quantitated, and then sequenced and analyzed. GO and Kyoto Encyclopedia of Genes and Genomes (KEGG) enrichment analyses were performed to determine the biological implications of the DEGs.

### Signature construction

A univariate COX proportional hazards regression analysis was performed to determine the prognostic value of m6A-related genes. Subsequently, a stepwise COX proportional hazards regression model was used to filter out the most predictive genes among the survival-related candidate markers (**Supplementary Table S1**). Four genes and their coefficients were chosen with the minimum criteria, and the risk score for the signature was calculated using the following formula:


$$Riskscore=Coef_{1}*x_{1}+Coef_{2}*x_{2}+\ldots +Coef_{n}*x_{n}$$

where *Coef_n_* and *x_n_* are the coefficient and the *z*-score-transformed relative expression value for every chosen gene, respectively. Finally, HNSCC patients were divided into high- and low-risk groups according the median risk score.

### Mouse xenograft model

All animal experiments were approved by the Animal Ethics Committee of the Zhengzhou Cancer Institute in Henan. Female BALB/c-nu/nu mice (6–8 weeks old) were bred at Zhengzhou Cancer Institute (Henan, China) and housed under specific pathogen-free (SPF) conditions in a laboratory animal facility. A total of 3.5 × 10^5^ wild-type CAL-27 and HSC-3 cells or those stably expressing shHNRNPC were subcutaneously injected into the right dorsal flank of the mice (5 per group). The mice weight and tumor size were measured once every three days. After 25 days, the mice were sacrificed, and the tumors were harvested at necropsy for further analysis.

### Statistical analysis

Pearson correlation analysis was performed to assess the association between two regulators. Kaplan–Meier curves were used to compare the OS or recurrence-free survival (RFS) rates of patients between the high- and low-risk groups, and the survival distributions in the two groups was evaluated by the log-rank test. The clinical characteristics were compared using the chi-square test. While differences between groups were compared using a Student’s *t*-test. Statistical analyses were performed using the R software version 3.6.1 (https://www.r-project.org), IBM SPSS Statistics V21.0 (IBM SPSS Inc., Chicago, USA), or GraphPad Prism 7.0 (GraphPad, San Diego, CA, USA). *P* < 0.05 was considered statistically significant.

## Results

### Establishment of the specific survival risk model of m6A-associated enzymes

**Figure 1:**
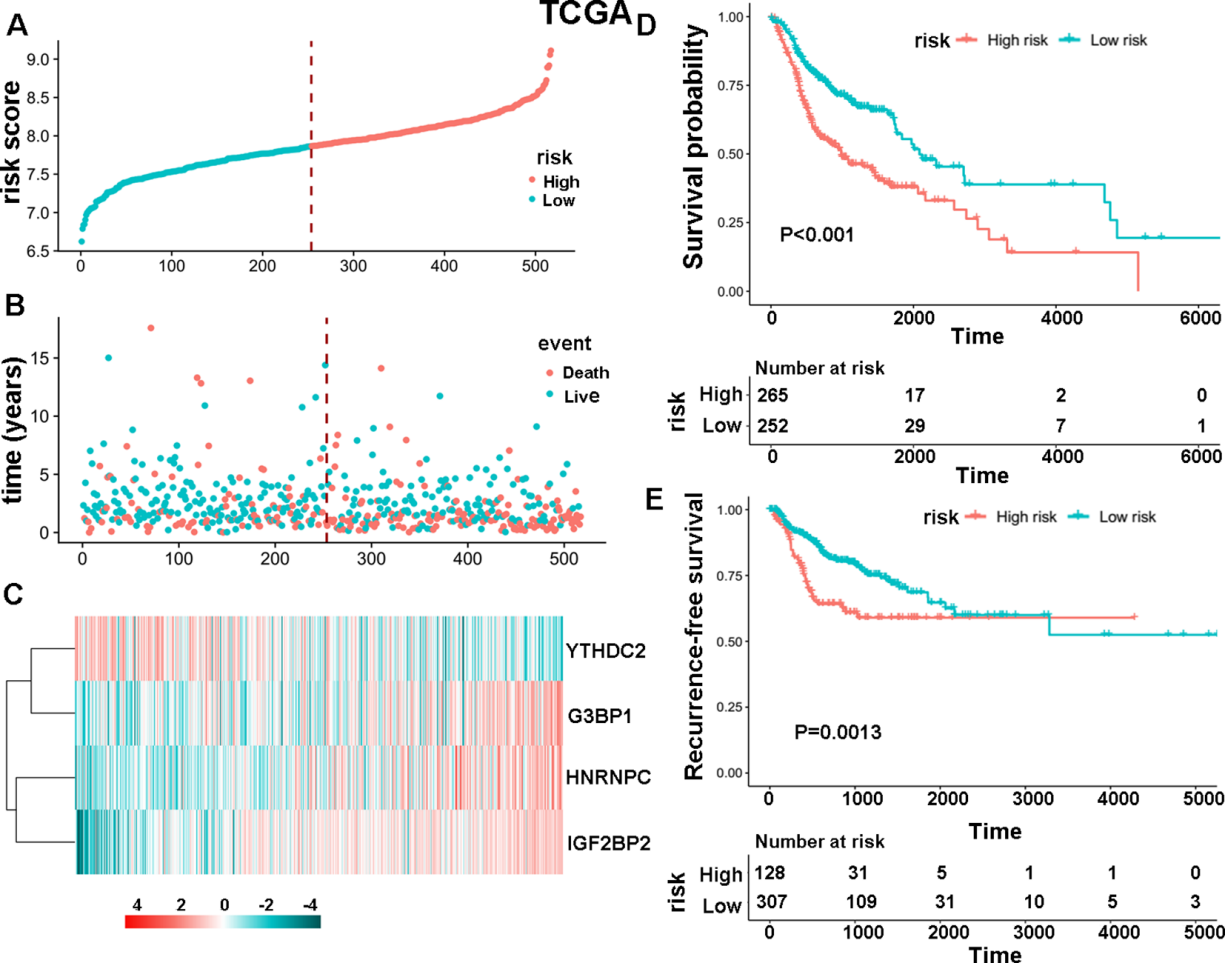
The specific survival risk model of m6A-associated enzymes. **(A)**The risk score distribution of 517 HNSCC patients. The blue and red dots indicate low and high scores, respectively. **(B)** Distribution of the survival status. The blue and red dots indicate alive and dead status, respectively. **(C)** Heatmap showing the gene expression levels of four m6A-associated enzymes: YTHDC2, G3BP1, HNRNPC and IGF2BP2. **(D, E)** Comparison of OS and RFS between high-risk and low-risk groups of patients.

The study initially analyzed the expression levels of 30 m6A regulators in 504 HNSCC samples and 44 normal tissues from the TCGA database, finding that 28 genes were upregulated and two were not significantly different (**Supplementary Figure S1**). Subsequently, data from 517 HNSCC patients was used to develop a specific survival risk model based on m6A-associated enzymes’ gene expression. This model identified a high-risk group of 265 patients and a low-risk group of 252 patients (**Figure 1A**). The survival status of these patients is shown in **Figure 1B**. Results of survival analysis based on these four m6A-associated proteins (**Figure 1C**) show that the HNSCC patients in the high-risk group had a significantly shorter OS (**Figure 1D**, *P* < 0.001) and recurrence-free survival (RFS) (**Figure 1E**, *P* = 0.0013) than those in the low-risk group. We further evaluated the survival status of both the high-risk and low-risk groups according to several clinicopathological parameters including clinical stage, status of lymph node metastasis, and gender. The results demonstrate that the high-risk patients had a poorer prognosis than the low-risk patients (**Supplementary Figure S2**). These findings were validated in an independent dataset from the GEO database, confirming the stability and reliability of the m6A-associated survival risk model. (**Supplementary Figures S3A-E**). Additionally, it was noted that *HNRNPC* showed the highest expression levels among the four selected genes in both HNSCC and paired normal tissues (**Supplementary Figure S3F**).

### HNSCC patients with high expression levels of *HNRNPC* have a worse prognosis

**Figure 2:**
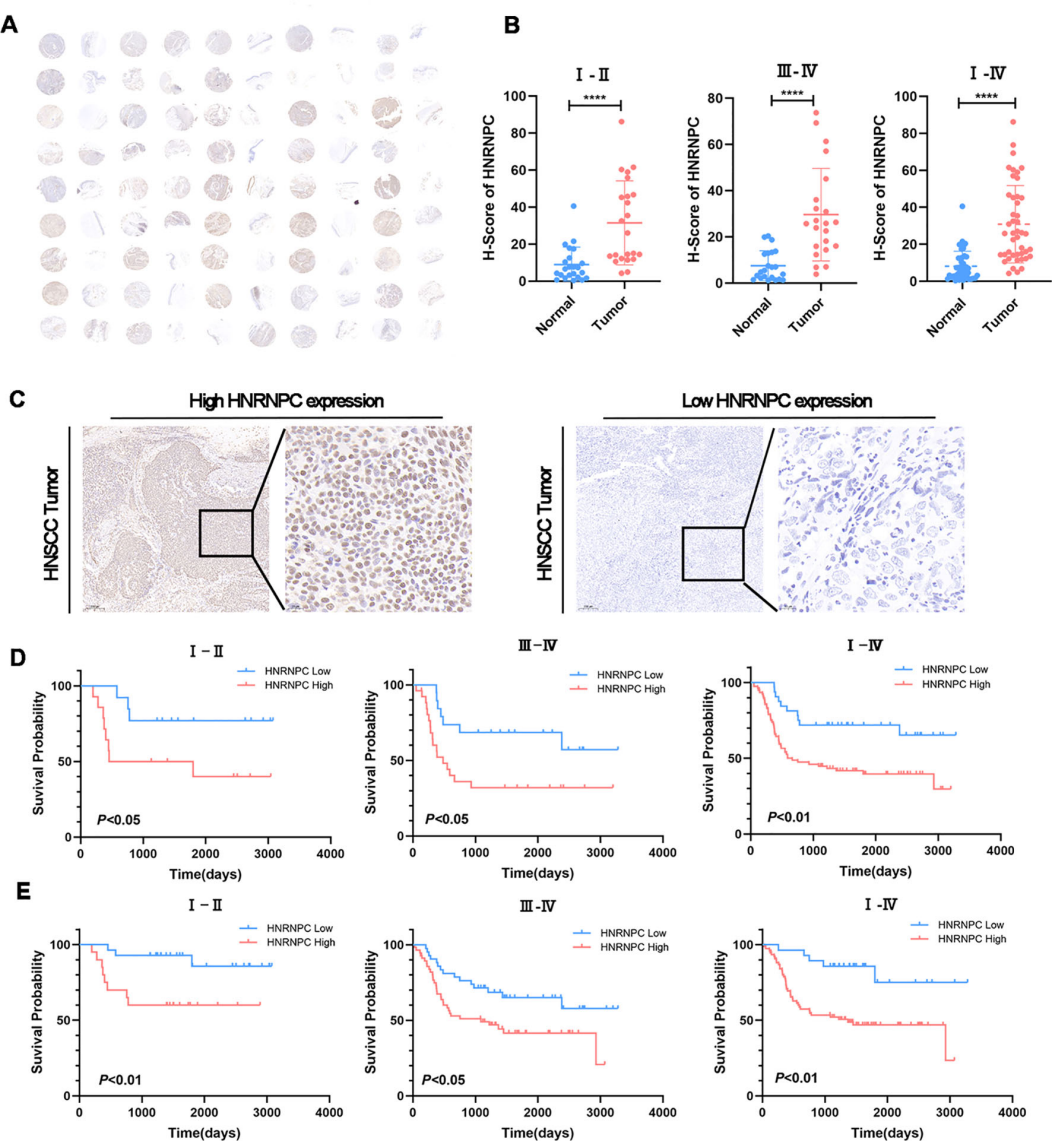
HNSCC patients with a high expression level of HNRNPC have a worse prognosis. **(A)** IHC staining for HNRNPC on TMA of HNSCC patients. Scale bar: 100 μm. **(B)** The expression levels of HNRNPC in TMA at stages I–II, III–IV, and I–IV. **(C)** Representative images of IHC staining for HNRNPC in squamous cell carcinoma tissue of the head and neck. Scale bar: 200 μm. **(D, E)** According to IHC or qPCR, Kaplan-Meier survival curves were constructed for HNSCC patients with high or low HNRNPC expression levels at stages I–II, III–IV, and I–IV. The log-rank test was used to compare survival rates. ****P≤ 0.0001.

The tissue microarray (TMA) immunohistochemistry (IHC) results revealed significantly higher *HNRNPC* expression in HNSCC tissues compared to adjacent normal tissues (**Figures 2A, B**, *P* < 0.0001). Additionally, 150 tumor samples were collected from HNSCC patients. We further compared the clinical characteristics between patient groups with high and low *HNRNPC* expression levels and observed statistically significant differences in tumor stage, lymph node metastasis, and survival state. However, no significant differences were detected among the other clinical features (**Table 1**). The patients were then categorized into high and low expression groups based on the results of IHC (**Figure 2C**) and qPCR. Notably, patients with high *HNRNPC* expression in stages I–II, III–IV, and I–IV showed a lower survival probability in comparison with those with low expression (**Figures 2D, E**).

### Repression of *HNRNPC* arrests malicious biological behavior of CAL-27 and HSC-3 cells

**Figure 3:**
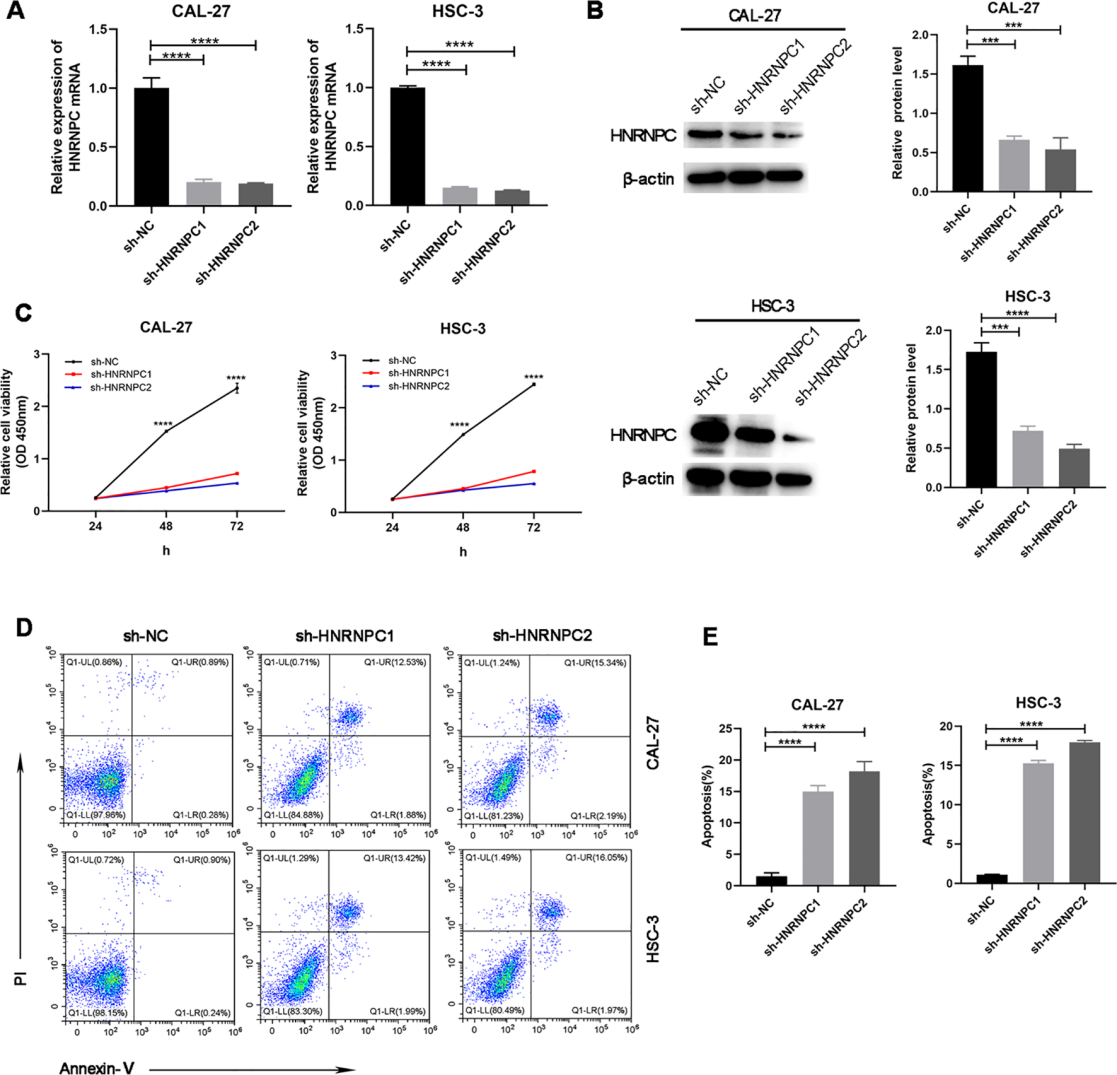
Knockdown of HNRNPC arrests proliferation and promotes apoptosis of CAL-27 and HSC-3 cells. **(A, B)** qPCR or immunoblotting of the relative expression levels of HNRNPC in CAL-27 and HSC-3 cells following knockdown of HNRNPC. **(C)** Proliferation rates of cells after knockdown of HNRNPC. **(D, E)** Flow cytometry was performed to analyze apoptosis. ***P≤ 0.001; ****P≤ 0.0001.

**Figure 4:**
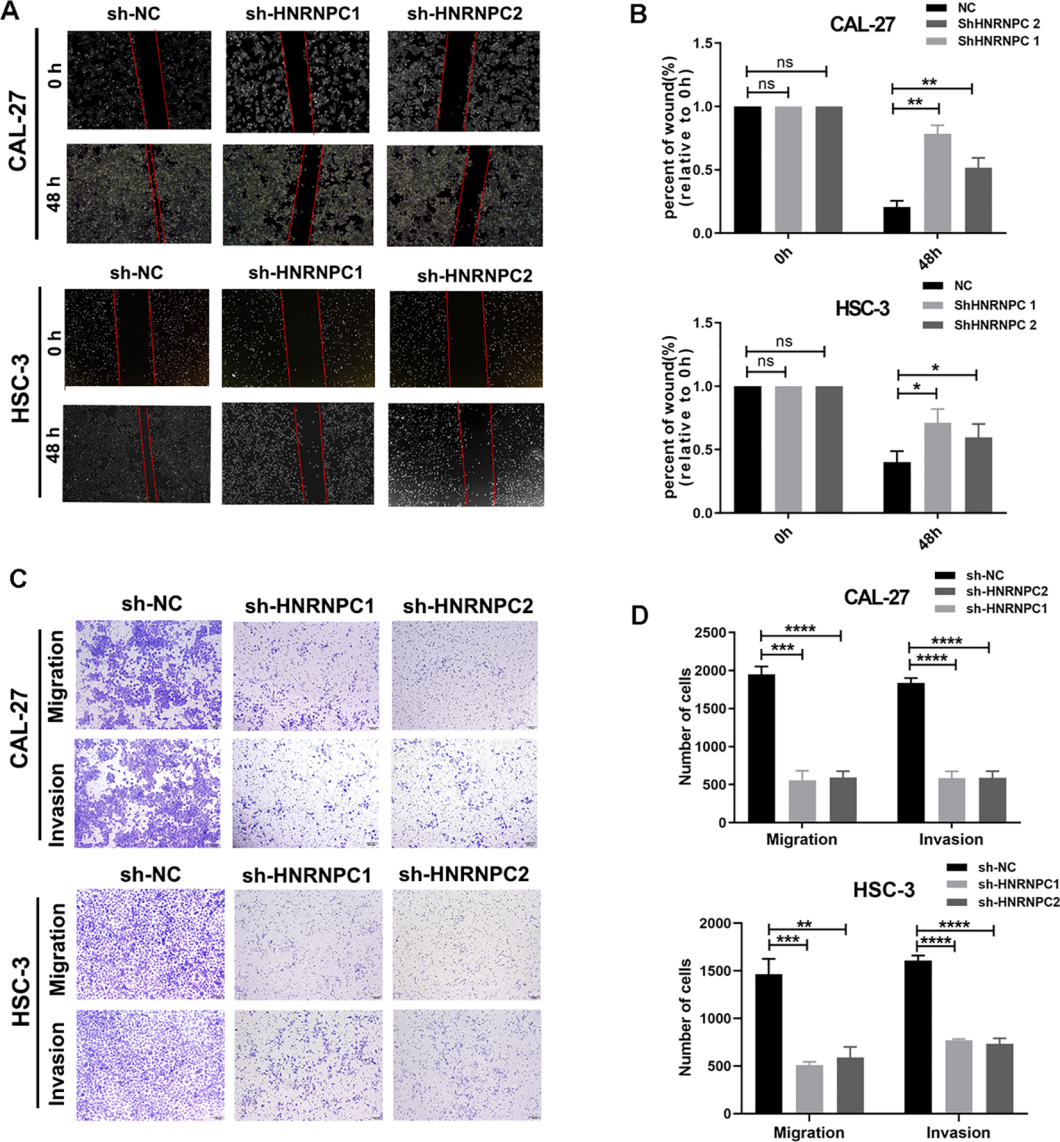
Knockdown of HNRNPC decreases the migratory and invasive abilities of CAL-27 and HSC-3 cells. **(A)** Images of the wound healing assay. **(B)** Wound area (relative to 0 h) was measured and analyzed. **(C)** Images of the migration and invasion assay. **(D)** The number of cells that migrated and invaded was recorded and analyzed. *P ≤ 0.05; **P≤ 0.01; ***P≤ 0.001; ****P≤ 0.0001; ns P>0.05.

Our study focused on the potential function of *HNRNPC* in HNSCC cells. The expression of *HNRNPC* in HSC-3 and CAL-27 cells was silenced by shRNA and verified at the protein and mRNA levels (**Figures 3A, B**). Knockdown of *HNRNPC* significantly reduced the cell proliferation rate at both 48 h and 72 h (**Figure 3C**, *P* < 0.001) and promoted apoptosis (**Figures 3D, E**). A larger wound area (**Figures 4A, B**) and a significantly reduced invasive ability (**Figures 4C, D**) were found following *HNRNPC* knockdown, strongly implying that *HNRNPC* plays a crucial role in the migration and invasion of CAL-27 and HSC-3 cells.

### *HNRNPC* is associated with cell differentiation, cell migration, cell cycle, cell proliferation, and apoptosis

**Figure 5:**
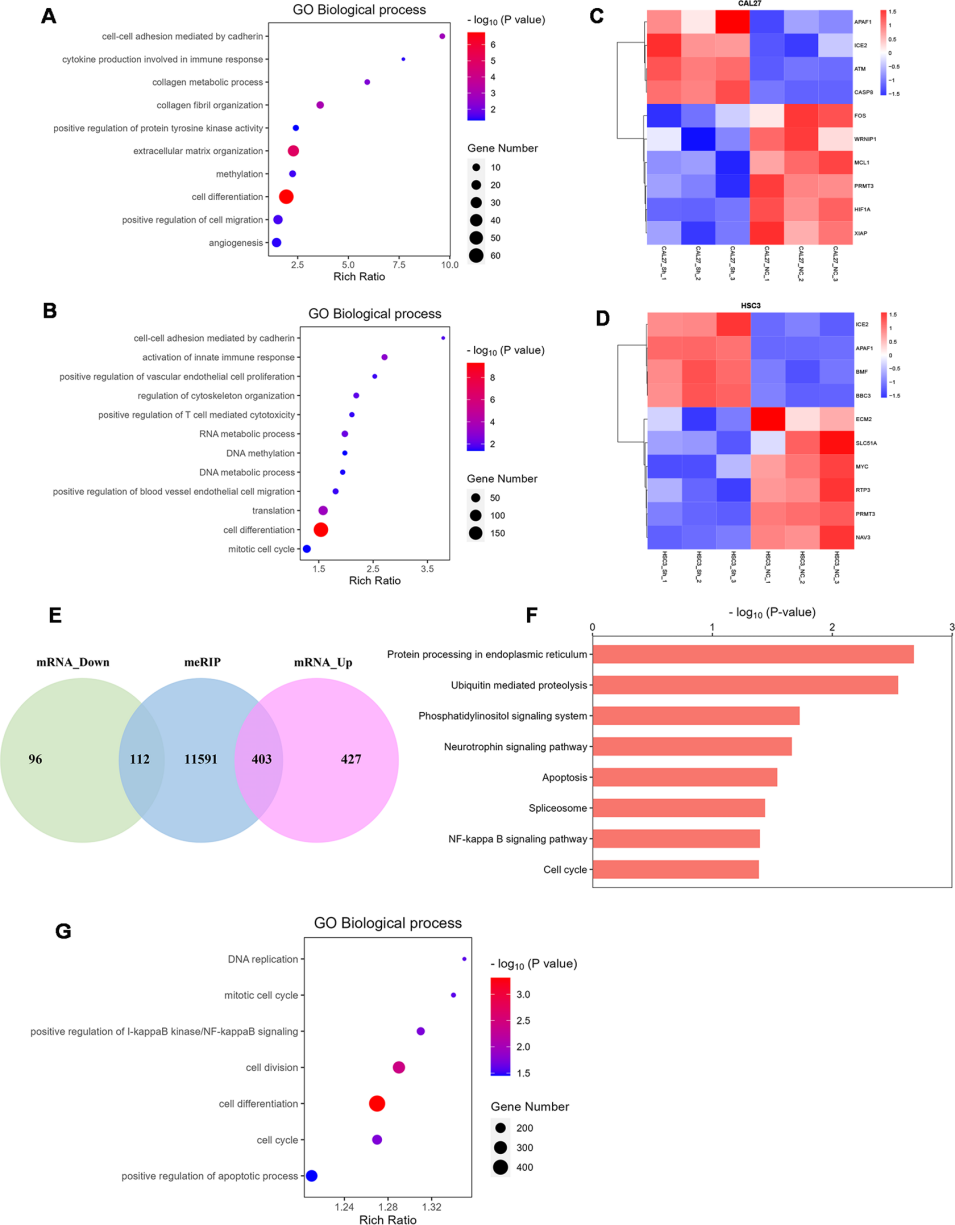
HNRNPC is associated with cell differentiation, cell migration, cell cycle, cell proliferation, and apoptosis. **(A, B)** Bubble chart demonstrating the typical GO terms associated with biological processes following the knockdown of HNRNPC in CAL-27 or HSC-3 cells. **(C, D)** Heatmap showing the genes with significant changes following HNRNPC knockdown in CAL-27 or HSC-3 cells (*P* < 0.05). **(E)** Venn diagram showing the differentially expressed genes in HNRNPC-knockdown CAL-27 cells. **(F)** Histogram depicting the enriched pathways identified by KEGG analysis of MeRIP-seq data following HNRNPC knockdown in CAL-27 cells. **(G)** Bubble chart showing the biological processes identified by MeRIP-seq analysis following HNRNPC knockdown in CAL-27 cells.

We knocked down *HNRNPC* in HSC-3 and CAL-27 cells and then performed RNA-seq. GO analysis identified that the differentially expressed genes (DEGs) were enriched in cell differentiation, cell migration, cell cycle, angiogenesis, extracellular matrix organization, cell proliferation, and T cell-mediated cytotoxicity (**Figures 5A, B**). DEGs involved in apoptosis, migration, and invasion are shown in the heatmap (**Figures 5C, D**). MeRIP-seq and conventional RNA-seq were combined to screen the DEGs in CAL-27 cells following knockdown of *HNRNPC* (**Figure 5E**). Results of KEGG analysis show that the DEGs were associated with tumor development processes, such as apoptosis and the cell cycle (**Figure 5F**), and GO analysis data demonstrate enrichment in apoptosis, cell differentiation, cell division, and the cell cycle (**Figure 5G**).

### HNRNPC promotes xenograft HNSCC tumor growth *in vivo*

**Figure 6:**
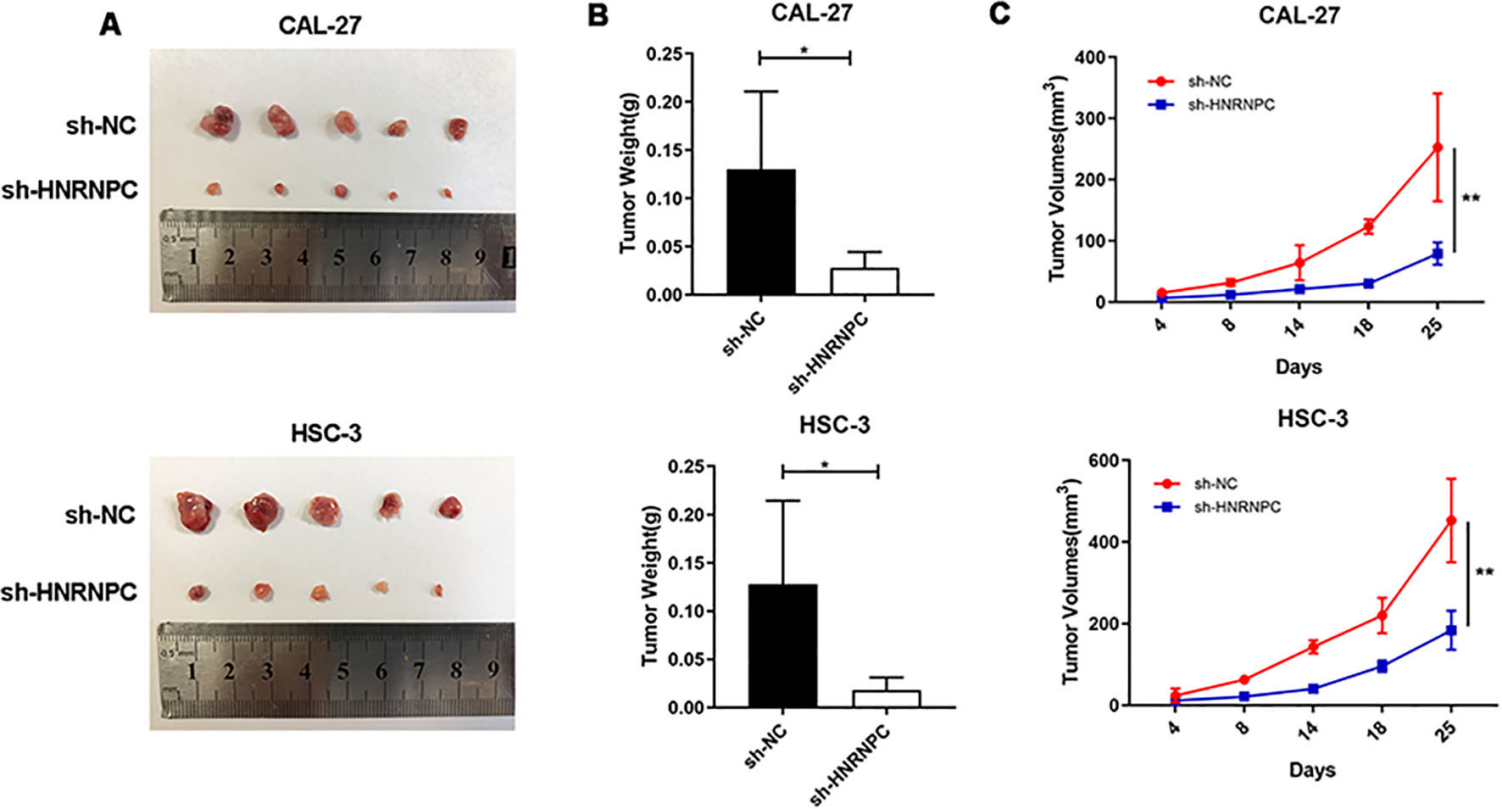
HNRNPC promotes HNSCC xenograft tumor growth *in vivo*. **(A)** HNRNPC-knockdown CAL-27 and HSC-3 cells were injected into mice. Representative photos of xenograft tumors are shown. **(B, C)** Weight and volume of HNRNPC-knockdown CAL-27 and HSC-3 tumors extracted from mice. *P ≤ 0.05; **P≤ 0.01.

To assess whether *HNRNPC* impacts tumorigenesis *in vivo*, a xenograft tumor model was established via subcutaneous injection of CAL-27-NC, CAL-27-shHNRNPC, HSC-3-NC, or HSC-3-shHNRNPC cells into the right dorsal flank of mice. After a period of 25 days, the mice were sacrificed and the tumors were surgically removed (**Figure 6A**). The tumor volume in the HNRNPC-knockdown groups was significantly lower (100 mm^3^ CAL-27 and 200 mm^3^ HSC-3) than that in the control groups (250 mm^3^ CAL-27 and 500 mm^3^ HSC-3) (*P <* 0.05) (**Figures 6B, C**).

## Discussion

The initiation and development of HNSCC is a multistep process that involves gradual acquisition of genetic and epigenetic alterations, leading to uncontrolled growth and proliferation of tumor cells (20). m6A methylation is the most abundant mRNA and long noncoding RNA (lncRNA) modification in mammals, occurring at the sixth N position of adenosine and being dynamic and reversible (21). Increasing evidence suggests that m6A methylation plays a critical role in malignant tumors. Aberrant m6A RNA methylation modifications have been demonstrated to facilitate carcinogenesis and the progression of many tumor types, such as bladder cancer (22), ovarian cancer (23), and hepatocellular carcinoma (24), in addition to contributing to the formation of the tumor microenvironment and poor prognosis in gastric (25), and lung cancer (26). Herein, m6A-related genes were extracted from the TCGA database and a risk score was constructed using a univariate and stepwise COX proportional hazards regression analysis. The risk score indicates that four genes, *YTHDC2*, *HNRNPC*, *IGF2BP2* and *G3BP1*, were associated with poor OS and RFS of HNSCC patients. Moreover, the risk score demonstrates a significant association with clinical parameters involved in tumor stage, lymph node metastasis and gender, which was further verified using GEO data. Consistent with the findings of this study, univariate Cox regression analyses of TCGA-HNSCC cohort data from three independent studies indicate that the expression of several m6A regulators is linked to clinical outcomes, including overall survival in HNSCC patients (27–29). Cai et al. found a strong positive correlation between m6A regulator expression and HNSCC progression. Their forest plot analysis of Cox regression data identified m6A readers, such as IGF2BP1, IGF2BP2, and *HNRNPC*, as factors associated with poor prognosis (29). Among the four genes selected in this study, *YTHDC2*, as a member of the YTH protein family, can recognize m6A-binding mRNA in the nucleus, improving translation efficiency and reducing mRNA abundance (30). Knockout of *YTHDC2* weakens the expression of metastasis-related genes, such as hypoxia-inducible factor-1 alpha (HIF-1α) and twist1, thereby inhibiting the metastasis of colon tumor cells (31). The prognosis is favorable for patients with downregulated *YTHDC2* expression in HNSCC (32). *IGF2BP2* is a member of the insulin-like growth factor-2 binding protein family, which promotes more stable mRNA translation in an m6A-dependent manner (11). *IGF2BP2* overexpression promotes pancreatic ductal adenocarcinoma progression by binding to and stabilizing glucose transporter 1 (GLUT1) mRNA (33). Analysis of the TCGA database and IHC demonstrate that *IGF2BP2* is overexpressed in HNSCC patients with a poor prognosis (34). *G3BP1* is a member of the Ras-GTPase-activating protein binding protein family and plays an important role in RNA metabolism (35). Knockdown of *G3BP1* inhibits the G0/G1 phase of the cell cycle and reduces cell proliferation, migration, and invasion by inactivating the PI3K/AKT/mTOR and Wnt signaling pathways in esophageal carcinoma (36). *G3BP1* promotes renal cell carcinoma by connecting IL-6 and STAT3 signal transduction, and increases CD4^+^ T cell infiltration during the progression and metastasis of oral squamous cell carcinoma (OSCC) (37).

Among the four selected genes, *HNRNPC* had the highest expression in both tumor and normal tissues in the TCGA database. Our findings indicate that *HNRNPC* is highly expressed in tumor tissues compared to adjacent normal tissues, aligning with previous studies that suggest a tumorigenic role for *HNRNPC* in HNSCC (38). However, further *in vitro* and *in vivo* investigation is required to harvest more accurate functional information for *HNRNPC* in HNSCC. Here, knockdown of *HNRNPC* significantly inhibited cell proliferation and promoted cell apoptosis *in vitro*, in addition to impairing tumorigenesis in the mouse model. Moreover, RNA-seq and MeRIP-seq data demonstrate that *HNRNPC* is involved in cell differentiation, cell migration, cell cycle, cell proliferation, and apoptosis. These findings demonstrate that *HNRNPC* plays an critical role in HNSCC tumorigenesis. To date, several studies have focused on the role of *HNRNPC* in tumorigenesis and development. An increasing number of reports demonstrate that *HNRNPC* plays distinct roles in the metastasis and invasion of different cancers; for instance, elevated levels of *HNRNPC* are essential for the proliferative ability of two breast cancer cell lines (39). Wang et al. found that *HNRNPC* expression was elevated in glioma tissues compared to normal brain tissues, and higher levels of *HNRNPC* were associated with advanced clinical stages (40). Furthermore, overexpression of *HNRNPC* promotes OSCC progression via epithelial–mesenchymal transition (EMT) (41), in addition to chemoresistance in gastric cancer with poor OS and free of progression (FP) (42).

Although the present study has important clinical significance, several limitations still need to be considered. To gain a comprehensive understanding of the molecular landscape of HNSCC and further refine the risk score model, it is essential to investigate the underlying mechanisms involving *HNRNPC*. Additionally, further studies are warranted to investigate the function and underlying mechanisms of the remaining three identified m6A biomarkers.

## Conclusions

The present study demonstrates that *HNRNPC* is involved in the development of HNSCC and patient prognosis. Knockdown of *HNRNPC* can inhibit proliferation and metastasis and promote apoptosis of HNSCC cells. *HNRNPC* may become a new biomarker for predicting the prognosis of HNSCC patients, providing new insights and tools for treatment.

## Data Availability

The original contributions presented in the study are included in the article/**Supplementary Material**. Further inquiries can be directed to the corresponding author/s.

## References

[1] Forastiere A Koch W Trotti A Sidransky D. Head and neck cancer. N Engl J Med. (2001) 345:1890–900. doi: 10.1056/NEJMra001375 11756581

[2] Sanderson RJ Ironside JA. Squamous cell carcinomas of the head and neck. BMJ. (2002) 325:822–7. doi: 10.1136/bmj.325.7368.822 PMC1124330 12376446

[3] Ludwig ML Birkeland AC Hoesli R Swiecicki P Spector ME Brenner JC. Changing the paradigm: the potential for targeted therapy in laryngeal squamous cell carcinoma. Cancer Biol Med. (2016) 13:87–100. doi: 10.20892/j.issn.2095-3941.2016.0010 27144065 PMC4850131

[4] He L Li H Wu A Peng Y Shu G Yin G. Functions of N6-methyladenosine and its role in cancer. Mol Cancer. (2019) 18:176. doi: 10.1186/s12943-019-1109-9 31801551 PMC6892141

[5] Jia G Fu Y He C. Reversible RNA adenosine methylation in biological regulation. Trends Genet. (2013) 29:108–15. doi: 10.1016/j.tig.2012.11.003 PMC3558665 23218460

[6] Liu J Yue Y Han D Wang X Fu Y Zhang L. A METTL3-METTL14 complex mediates mammalian nuclear RNA N6-adenosine methylation. Nat Chem Biol. (2014) 10:93–5. doi: 10.1038/nchembio.1432 PMC3911877 24316715

[7] Jia G Fu Y Zhao X Dai Q Zheng G Yang Y. N6-methyladenosine in nuclear RNA is a major substrate of the obesity-associated FTO. Nat Chem Biol. (2011) 7:885–7. doi: 10.1038/nchembio.687 PMC3218240 22002720

[8] Li A Chen YS Ping XL Yang X Xiao W Yang Y. Cytoplasmic m(6)A reader YTHDF3 promotes mRNA translation. Cell Res. (2017) 27:444–7. doi: 10.1038/cr.2017.10 PMC5339832 28106076

[9] Ma S Chen C Ji X Liu J Zhou Q Wang G. The interplay between m6A RNA methylation and noncoding RNA in cancer. J Hematol Oncol. (2019) 12:121. doi: 10.1186/s13045-019-0805-7 31757221 PMC6874823

[10] König J Zarnack K Rot G Curk T Kayikci M Zupan B. iCLIP reveals the function of hnRNP particles in splicing at individual nucleotide resolution. Nat Struct Mol Biol. (2010) 17:909–15. doi: 10.1038/nsmb.1838 PMC3000544 20601959

[11] Huang H Weng H Sun W Qin X Shi H Wu H. Recognition of RNA N(6)-methyladenosine by IGF2BP proteins enhances mRNA stability and translation. Nat Cell Biol. (2018) 20:285–95. doi: 10.1038/s41556-018-0045-z PMC5826585 29476152

[12] Zarnack K König J Tajnik M Martincorena I Eustermann S Stévant I. Direct competition between hnRNP C and U2AF65 protects the transcriptome from the exonization of Alu elements. Cell. (2013) 152:453–66. doi: 10.1016/j.cell.2012.12.023 PMC3629564 23374342

[13] McCloskey A Taniguchi I Shinmyozu K Ohno M. hnRNP C tetramer measures RNA length to classify RNA polymerase II transcripts for export. Science. (2012) 335:1643–6. doi: 10.1126/science.1218469 22461616

[14] Velusamy T Shetty P Bhandary YP Liu MC Shetty S. Posttranscriptional regulation of urokinase receptor expression by heterogeneous nuclear ribonuclear protein C. Biochemistry. (2008) 47:6508–17. doi: 10.1021/bi702338y 18494499

[15] Gruber AJ Schmidt R Gruber AR Martin G Ghosh S Belmadani M. A comprehensive analysis of 3’ end sequencing data sets reveals novel polyadenylation signals and the repressive role of heterogeneous ribonucleoprotein C on cleavage and polyadenylation. Genome Res. (2016) 26:1145–59. doi: 10.1101/gr.202432.115 PMC4971764 27382025

[16] Lee EK Kim HH Kuwano Y Abdelmohsen K Srikantan S Subaran SS. hnRNP C promotes APP translation by competing with FMRP for APP mRNA recruitment to P bodies. Nat Struct Mol Biol. (2010) 17:732–9. doi: 10.1038/nsmb.1815 PMC2908492 20473314

[17] Park YM Hwang SJ Masuda K Choi KM Jeong MR Nam DH. Heterogeneous nuclear ribonucleoprotein C1/C2 controls the metastatic potential of glioblastoma by regulating PDCD4. Mol Cell Biol. (2012) 32:4237–44. doi: 10.1128/MCB.00443-12 PMC3457347 22907752

[18] Liu J Sun G Pan S Qin M Ouyang R Li Z. The Cancer Genome Atlas (TCGA) based m(6)A methylation-related genes predict prognosis in hepatocellular carcinoma. Bioengineered. (2020) 11:759–68. doi: 10.1080/21655979.2020.1787764 PMC8291839 32631107

[19] Sarbanes SL Le Pen J Rice CM. Friend and foe, HNRNPC takes on immunostimulatory RNAs in breast cancer cells. EMBO J. (2018) 37(23):e100923. doi: 10.15252/embj.2018100923 30389667 PMC6276875

[20] Johnson DE Burtness B Leemans CR Lui VWY Bauman JE Grandis JR. Head and neck squamous cell carcinoma. Nat Rev Dis Primers. (2020) 6:92. doi: 10.1038/s41572-020-00224-3 33243986 PMC7944998

[21] Gonzales-van-Horn SR Sarnow P. Making the mark: the role of adenosine modifications in the life cycle of RNA viruses. Cell Host Microbe. (2017) 21:661–9. doi: 10.1016/j.chom.2017.05.008 PMC5555051 28618265

[22] Han J Wang JZ Yang X Yu H Zhou R Lu HC. METTL3 promote tumor proliferation of bladder cancer by accelerating pri-miR221/222 maturation in m6A-dependent manner. Mol Cancer. (2019) 18:110. doi: 10.1186/s12943-019-1036-9 31228940 PMC6588935

[23] Liu T Wei Q Jin J Luo Q Liu Y Yang Y. The m6A reader YTHDF1 promotes ovarian cancer progression via augmenting EIF3C translation. Nucleic Acids Res. (2020) 48:3816–31. doi: 10.1093/nar/gkaa048 PMC7144925 31996915

[24] Chen Y Peng C Chen J Chen D Yang B He B. WTAP facilitates progression of hepatocellular carcinoma via m6A-HuR-dependent epigenetic silencing of ETS1. Mol Cancer. (2019) 18:127. doi: 10.1186/s12943-019-1053-8 31438961 PMC6704583

[25] Zhang B Wu Q Li B Wang D Wang L Zhou YL. m(6)A regulator-mediated methylation modification patterns and tumor microenvironment infiltration characterization in gastric cancer. Mol Cancer. (2020) 19:53. doi: 10.1186/s12943-020-01170-0 32164750 PMC7066851

[26] Li Y Gu J Xu F Zhu Q Chen Y Ge D. Molecular characterization, biological function, tumor microenvironment association and clinical significance of m6A regulators in lung adenocarcinoma. Brief Bioinform. (2021) 22(4):bbaa225. doi: 10.1093/bib/bbaa225 33003204

[27] Chen Y Jiang X Li X Yan D Liu J Yang J. The methylation modification of m6A regulators contributes to the prognosis of head and neck squamous cell carcinoma. Ann Transl Med. (2021) 9:1346. doi: 10.21037/atm-21-4077 34532483 PMC8422133

[28] Zhai Y Zheng L. m6A RNA methylation regulator-related signatures exhibit good prognosis prediction ability for head and neck squamous cell carcinoma. Sci Rep. (2022) 12:16358. doi: 10.1038/s41598-022-20873-6 36175504 PMC9523032

[29] Cai H Liang J Jiang Y Tan R Hou C Hou J. Integrative analysis of N6-methyladenosine-related enhancer RNAs identifies distinct prognosis and tumor immune micro-environment patterns in head and neck squamous cell carcinoma. Cancers (Basel). (2022) 14(19):4657. doi: 10.3390/cancers14194657 36230580 PMC9563840

[30] Hsu PJ Zhu Y Ma H Guo Y Shi X Liu Y. Ythdc2 is an N(6)-methyladenosine binding protein that regulates mammalian spermatogenesis. Cell Res. (2017) 27:1115–27. doi: 10.1038/cr.2017.99 PMC5587856 28809393

[31] Tanabe A Tanikawa K Tsunetomi M Takai K Ikeda H Konno J. RNA helicase YTHDC2 promotes cancer metastasis via the enhancement of the efficiency by which HIF-1α mRNA is translated. Cancer Lett. (2016) 376:34–42. doi: 10.1016/j.canlet.2016.02.022 26996300

[32] Kasowitz SD Ma J Anderson SJ Leu NA Xu Y Gregory BD. Nuclear m6A reader YTHDC1 regulates alternative polyadenylation and splicing during mouse oocyte development. PLoS Genet. (2018) 14:e1007412. doi: 10.1371/journal.pgen.1007412 29799838 PMC5991768

[33] Huang S Wu Z Cheng Y Wei W Hao L. Insulin-like growth factor 2 mRNA binding protein 2 promotes aerobic glycolysis and cell proliferation in pancreatic ductal adenocarcinoma via stabilizing GLUT1 mRNA. Acta Biochim Biophys Sin (Shanghai). (2019) 51:743–52. doi: 10.1093/abbs/gmz048 31089713

[34] Deng X Jiang Q Liu Z Chen W. Clinical significance of an m6A reader gene, IGF2BP2, in head and neck squamous cell carcinoma. Front Mol Biosci. (2020) 7:68. doi: 10.3389/fmolb.2020.00068 32391379 PMC7193208

[35] Sahoo PK Lee SJ Jaiswal PB Alber S Kar AN Miller-Randolph S. Axonal G3BP1 stress granule protein limits axonal mRNA translation and nerve regeneration. Nat Commun. (2018) 9:3358. doi: 10.1038/s41467-018-05647-x 30135423 PMC6105716

[36] Zhang LN Zhao L Yan XL Huang YH. Loss of G3BP1 suppresses proliferation, migration, and invasion of esophageal cancer cells via Wnt/β-catenin and PI3K/AKT signaling pathways. J Cell Physiol. (2019) 234:20469–84. doi: 10.1002/jcp.v234.11 30989663

[37] Hu X Xia K Xiong H Su T. G3BP1 may serve as a potential biomarker of proliferation, apoptosis, and prognosis in oral squamous cell carcinoma. J Oral Pathol Med. (2021) 50:995–1004. doi: 10.1111/jop.v50.10 33987877

[38] Zhao X Cui L. Development and validation of a m(6)A RNA methylation regulators-based signature for predicting the prognosis of head and neck squamous cell carcinoma. Am J Cancer Res. (2019) 9:2156–69. PMC6834477 31720080

[39] Wu Y Zhao W Liu Y Tan X Li X Zou Q. Function of HNRNPC in breast cancer cells by controlling the dsRNA-induced interferon response. EMBO J. (2018) 37(23):e99017. doi: 10.15252/embj.201899017 30158112 PMC6276880

[40] Wang LC Chen SH Shen XL Li DC Liu HY Ji YL. M6A RNA methylation regulator HNRNPC contributes to tumorigenesis and predicts prognosis in glioblastoma multiforme. Front Oncol. (2020) 10:536875. doi: 10.3389/fonc.2020.536875 33134160 PMC7578363

[41] Huang GZ Wu QQ Zheng ZN Shao TR Chen YC Zeng WS. M6A-related bioinformatics analysis reveals that HNRNPC facilitates progression of OSCC via EMT. Aging (Albany NY). (2020) 12:11667–84. doi: 10.18632/aging.103333 PMC7343469 32526707

[42] Huang H Han Y Zhang C Wu J Feng J Qu L. HNRNPC as a candidate biomarker for chemoresistance in gastric cancer. Tumour Biol. (2016) 37:3527–34. doi: 10.1007/s13277-015-4144-1 26453116

