# Supplementary material for: Expression and prognostic significance of the m6A RNA methylation regulator HNRNPC in HNSCC
Source: Front Oncol. 2025 Feb 7;15:1516867. doi: 10.3389/fonc.2025.1516867 (PMC11842334; doi:10.3389/fonc.2025.1516867)
Supplement: Supplementary file 1 [file DataSheet1.docx]

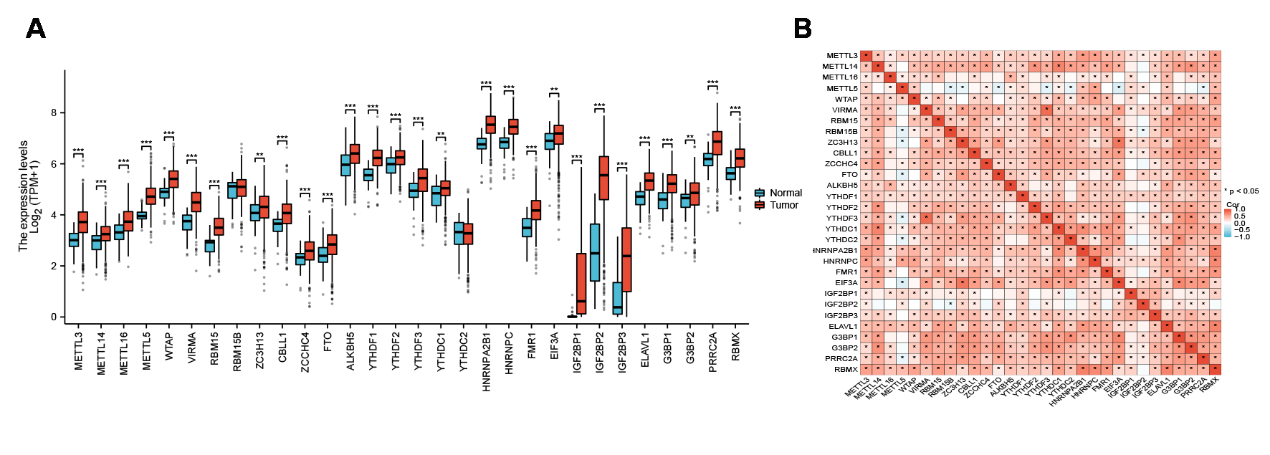


FIGURE S1. Expression levels of m6A RNA methylation regulators in HNSCC. (A) The expression levels of 30 m6A RNA methylation regulators in HNSCC. Tumor, red; Normal, blue. (B)Pearson correlation analysis of the relationship among m6A RNA methylation regulators.


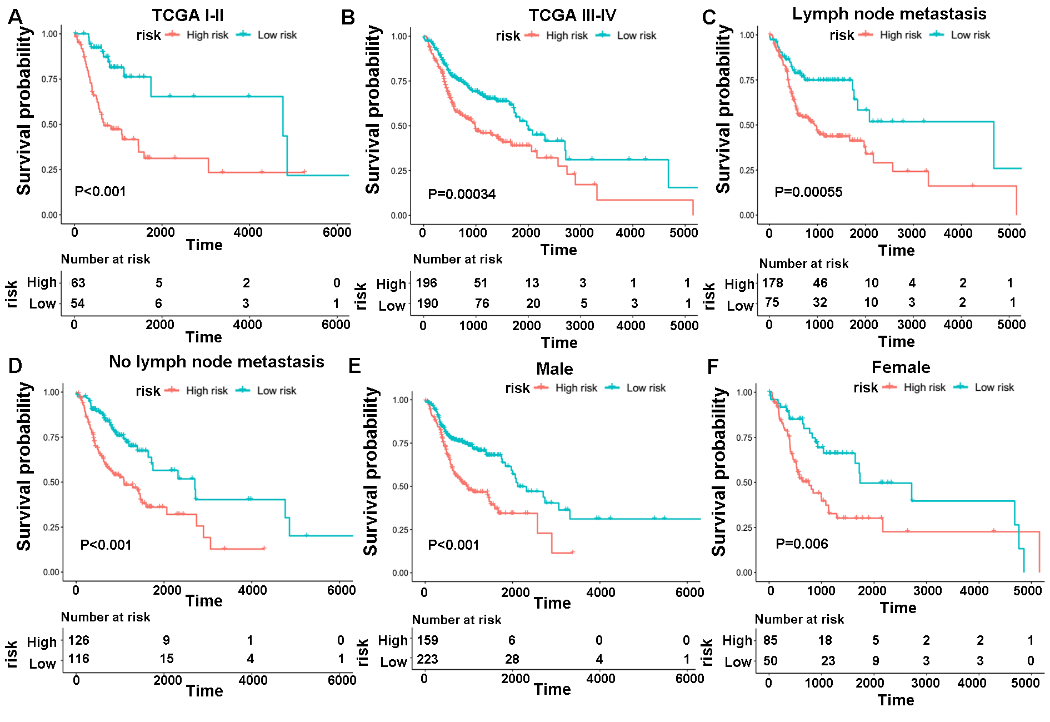


FIGURE S2. Prognostic significance of the m6A-specific survival risk model in the clinical subgroup of HNSCC patients. (A, B) The difference in OS between the high-risk and low-risk groups as stratified by stage. (C, D) The difference in OS between the high-risk and low-risk groups as stratified by lymph node metastasis.(E, F) The difference in OS between the high-risk and low-risk groups as stratified by gender.


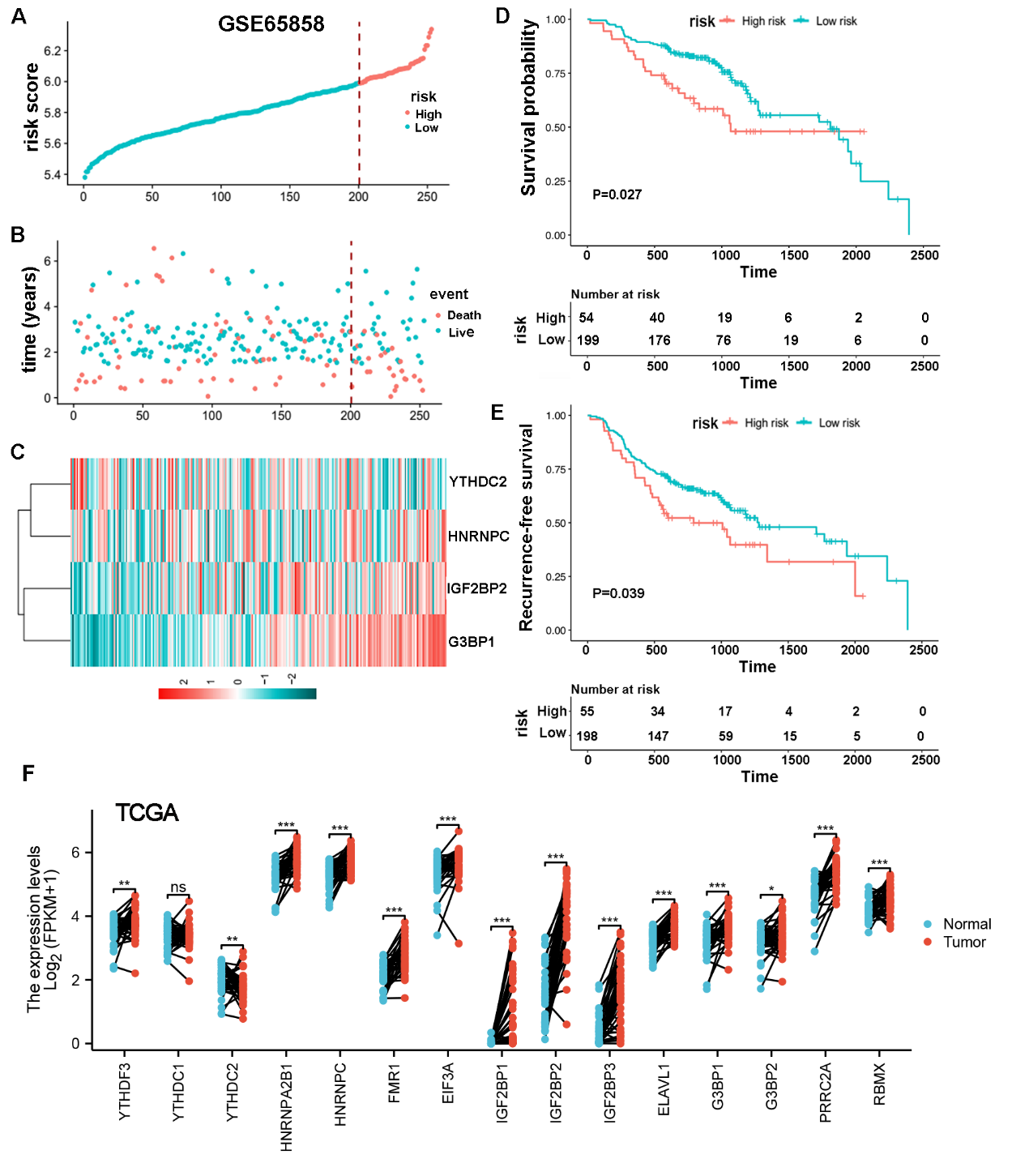


FIGURE S3. Validation of the m6A-specific survival risk model. (A) Distribution of the risk score. (B) Distribution of the survival status. The blue and red dots indicate alive and dead status, respectively. (C) Heatmap showing the gene expression levels of four m6A-associated enzymes. (D, E) OS or RFS of the high-risk group and the low-risk group. (F) The expression of 15 m6A RNA methylation regulators in paired HNSCC samples from the TCGA database. Tumor, red; Normal, blue.
