# Supplementary material for: Expression and prognostic significance of the m6A RNA methylation regulator HNRNPC in HNSCC
Source: Front Oncol. 2025 Feb 7;15:1516867. doi: 10.3389/fonc.2025.1516867 (PMC11842334; doi:10.3389/fonc.2025.1516867)
Supplement: Supplementary file 2 [file Table1.docx]

Table S1：Stepwise COX proportional hazards regression model

| Gene symbol | Coeff^*^ | Type^#^ | HR | lower95 | upper95 | *P*-value^$^ |
| --- | --- | --- | --- | --- | --- | --- |
| YTHDC2 | -0.4160 | protective | 0.6596 | 0.5118 | 0.8499 | 0.0013 |
| HNRNPC | 0.2990 | risky | 1.3485 | 0.8978 | 2.0253 | 0.1495 |
| IGF2BP2 | 0.0936 | risky | 1.0981 | 0.9938 | 1.2134 | 0.0660 |
| G3BP1 | 0.5870 | risky | 1.7986 | 1.2578 | 2.5720 | 0.0012 |
| HR, hazard ratio. | | | |  |  |  |
| ^#^ Types included protective (low risk) and risky (high risk). | | | | | |  |
| ^*^Coefficents derived from multivariable Cox regression analysis. | | | | | | |
| ^$^*P*-values obtained from multivariable Cox regression analysis. | | | | | |  |
