# Supplementary material for: Expression and prognostic significance of the m6A RNA methylation regulator HNRNPC in HNSCC
Source: Front Oncol. 2025 Feb 7;15:1516867. doi: 10.3389/fonc.2025.1516867 (PMC11842334; doi:10.3389/fonc.2025.1516867)
Supplement: Supplementary file 3 [file Table2.docx]

**Table S2：The REMARK checklist**

| **INTRODUCTION**  1 Utilizing the Cancer Genome Atlas (TCGA) database, the expression levels of m6A regulators in HNSCC were examined, which constructed a survival risk model. |
| --- |
| **MATERIALS AND METHODS**  Patients  2 Level 3 TCGA RNA-seq data for 517 HNSCC samples (Illumina® HiSeq 2000), with clinical annotations and overall survival (OS) information acquired from the Cancer Genomics Browser of University of California Santa Cruz (UCSC).  3 A total of 253 primary cases, also with clinical annotations and OS information, were selected from the GSE65858 dataset  Study design  4 A univariate COX proportional hazards regression analysis was performed to determine the prognostic value of m6A-related genes. Subsequently, a stepwise COX proportional hazards regression model was used to filter out the most predictive genes among the survival-related candidate markers.  5 Four genes and their coefficients were chosen with the minimum criteria, and the risk score for the signature was calculated using the following formula: *Riskscore = Coef1 *x1 + Coef2 *x2 + ...+Coefn *xn,* where Coefn and xn are the coefficient and the z-score-transformed relative expression value for every chosen gene, respectively.  6 HNSCC patients were divided into high- and low-risk groups according the median risk score. |
| **RESULTS**  7 This model identified a high-risk group of 265 patients and a low-risk group of 252 patients. Results of survival analysis based on these four m6A-associated proteins show that the HNSCC patients in the high-risk group had a significantly shorter OS (P < 0.001) and recurrence-free survival (RFS) (P = 0.0013) than those in the low-risk group.  8 We further evaluated the survival status of both the high-risk and low-risk groups according to several clinicopathological parameters including clinical stage, status of lymph node metastasis, and gender. The results demonstrate that the high-risk patients had a poorer prognosis than the low-risk patients.  9 These findings were validated in an independent dataset from the GEO database, confirming the stability and reliability of the m6A-associated survival risk model. |
| **DISCUSSION**  10 The risk score indicates that four genes, YTHDC2, HNRNPC, IGF2BP2 and G3BP1, were associated with poor OS and RFS of HNSCC patients. Moreover, the risk score demonstrates a significant association with clinical parameters involved in tumor stage, lymph node metastasis and gender, which was further verified using GEO data. |
